# Once-Weekly Insulin Icodec vs. Once-Daily Insulin Glargine U100 for type 2 diabetes: a systematic review and meta-analysis of phase 2 randomized controlled trials

**DOI:** 10.20945/2359-3997000000614

**Published:** 2023-05-29

**Authors:** Rodrigo Ribeiro e Silva, Mateus de Miranda Gauza, Maria Eduarda Schramm Guisso, Júlia Opolski Nunes da Silva, Suely Keiko Kohara

**Affiliations:** 1 Universidade da Região de Joinville Departamento de Medicina Joinville SC Brasil Departamento de Medicina, Universidade da Região de Joinville, Joinville, SC, Brasil

**Keywords:** Type 2 diabetes mellitus, insulin, insulin long-acting, insulin glargine, insulin icodec, glycemic control, glycated hemoglobin A

## Abstract

**Objective:**

Insulin Icodec is a novel basal insulin analogue designed for once-weekly administration, therefore might propitiate reduction in the frequency of injections and facilitate treatment adherence. This study aimed to determine the glycemic control and safety profile of Insulin Icodec, compared with Glargine U100 in patients with diabetes mellitus type 2.

**Materials and methods:**

We performed a systematic review and meta-analysis of randomized controlled trials (RCT) data comparing Once-Weekly Insulin Icodec and Once-Daily Insulin Glargine U100 in patients with type 2 diabetes mellitus. PubMed, Embase, and Cochrane databases were searched for trials published up to May 14, 2022. Data were extracted from published reports and quality assessment was performed per Cochrane recommendations.

**Results:**

Three studies were included comprising 453 patients, 230 (50.77%) using Once-Weekly Insulin Icodec and 223 (49.22%) using Once-Daily Insulin Glargine U100. In the pooled data, Glycated Hemoglobin (MD -0.20% CI -0.33 to -0.07%; P=0.002) change from baseline demonstrated a significantly higher reduction in the Icodec group. Time with Glucose in Range (MD 6.60% CI 3.63 to 9.57%; P < 0.0001) and Insulin Dose Difference (MD 0.97UI CI 0.76 to 1.18UI; P < 0.0001) were higher in the Icodec group. There was no significant difference in fasting plasma glucose, body weight change, hypoglycemia or any adverse event evaluated.

**Conclusions:**

Once-Weekly Insulin Icodec was associated with a small reduction in Glycated Hemoglobin, as well as higher Time with Glucose in Range, with similar hypoglycemic adverse events, when compared with Once-Daily Insulin Glargine U100.

## INTRODUCTION

Diabetes is a chronic and progressive illness requiring multiple strategies of interventions in order to reduce its burden (1). The progressive characteristic of the disease is clinically evidenced by the common need for optimization of pharmacological therapy, multiple times requiring the use of an external Insulin source. Insulin therapy in diabetes is a major cornerstone on its treatment, but the need for multiple daily subcutaneous applications and the risk of hypoglycemia becomes a challenge regarding patient adherence.

The necessity for optimizing treatment and reducing daily applications lead to the development of multiple insulin preparations and analogs in the past years, ranging from the first generation analogs, detemir and glargine-U100, to the second generation, glargine-U300 and degludec (2). Recently, a very innovative once weekly applied Insulin preparation was developed (3) and tested in a sequence of 3 phase 2 clinical trials (4-6), raising the expectations on this new type of treatment. Its use could help improve patient’s adherence to treatment as well as patient’s quality of life. However, an important issue that must be analyzed is effectiveness whether the new weekly applied insulin increases or diminishes the risk of hypoglycemia. Furthermore, several other phase 3 randomized clinical trials have started since, but there is no meta-analysis published on this subject to this date.

In light of this issue, we performed a systematic review and meta-analysis accessing the efficacy and safety of the weekly use of insulin Icodec in comparison to the daily insulin glargine, exploring populations with type 2 diabetes mellitus and their glycemic control.

## MATERIALS AND METHODS

### Eligibility criteria

Inclusion in this meta-analysis was restricted to studies that met all the following criteria: (1) randomized trials; (2) comparing the use once weekly insulin icodec to once daily insulin glargine; (3) enrolling patients with type 2 diabetes mellitus; (4) evaluating any of the desired outcomes; (5) articles written on English language. We excluded studies with (1) no control group; (2) overlapping studies population; clinical trial register entry only; (3) non-human studies and (4) studies reported only as abstracts.

### Search strategy

We systematically searched PubMed, Embase and Cochrane central register of controlled trials from inception to May 2022 with the following search term: “Icodec”. The references from all included studies were also searched manually for any additional studies. Two authors (M.M.G and M.E.G) independently screened and extracted the data following predefined search criteria and quality assessment. The prospective meta-analysis protocol was registered on INPLASY with registration number INPLASY202250102 (DOI number: 10.37766/inplasy2022.5.0102). The systematic review was registered after the beginning of the review process.

### Outcomes evaluated

The primary outcome was glycated hemoglobin (HbA1c). Outcomes included any adverse outcome, serious adverse event, any adverse event probably related to basal insulin, injection site reaction, hypersensitivity reaction, hypoglycemia alert, clinically significant or severe hypoglycemia, HBa1C (%) change from baseline, fasting plasma glucose (mg/dL) change from baseline, body weight (kg) change from baseline, time with glucose in range (%) and insulin dose difference (UI).

Time with Glucose in Range (TIR) varied between studies. Rosenstock (4) used a tight range (70-140 mg/dL), whereas Lingvay (5) and Bajaj (6) adopted 70-180 mg/dL. Percent TIR was calculated in the last two weeks of the trials, measured by continuous glucose measurement systems. Hypoglycemia alert (Level 1) was defined as Plasma Glucose < 70 mg/dL and > 54 mg/dL. While, clinically significant hypoglycemia determined as Plasma Glucose < 54 mg/dL and severe hypoglycemia as severe cognitive impairment requiring external assistance.

### Risk of bias assessment

We evaluated the risk of bias in randomized studies using version 2 of the Cochrane Risk of Bias assessment tool (7). Two independent authors completed the risk of bias assessment (R.R.S and J.O.N.S). Disagreements were resolved through a consensus after discussing reasons for discrepancies. Each trial received a score of high, low, or unclear risk of bias in 5 domains: selection, performance, detection, attrition, and reporting biases. All included studies contributed to the evaluated outcomes through forest plots. Small-study effect was investigated by funnel-plot analysis of point estimates in relation to study weights.

The certainty of evidence was classified according to the Grading of recommendation Assessment, development and Evaluation (GRADE) method. The systematic review and meta-analysis were performed and reported in accordance with the Cochrane Collaboration Handbook for Systematic Review of Interventions and the Preferred Reporting Items for Systematic Reviews and Meta-analysis (PRISMA) statement guidelines (8,9).

### Data analysis

Review Manager 5.3 (Cochrane Center, The Cochrane Collaboration, Denmark) was used for statistical analysis. Odds-ratios (OR) with 95% confidence intervals were used to compare treatment effect for categorical endpoints. Continuous outcomes were compared with mean differences. We assessed heterogeneity with I² statistics and Cochran Q test; p-values < 0.1 and I² > 25% were considered significant for heterogeneity. We used a fixed-effect model for outcomes with low heterogeneity (I² <2 5%). Otherwise, a DerSimonian and Laird random-effects model was used. We also performed sensitivity analyses by excluding individual studies to evaluate the impact of a single study in each outcome. No missing results were identified during data extraction.

### Role of funding source

There was no funding source for this study. All the authors had full access to all data in the study. The corresponding author had final responsibility for the decision to submit for publication.

## RESULTS

**Figure 1:**
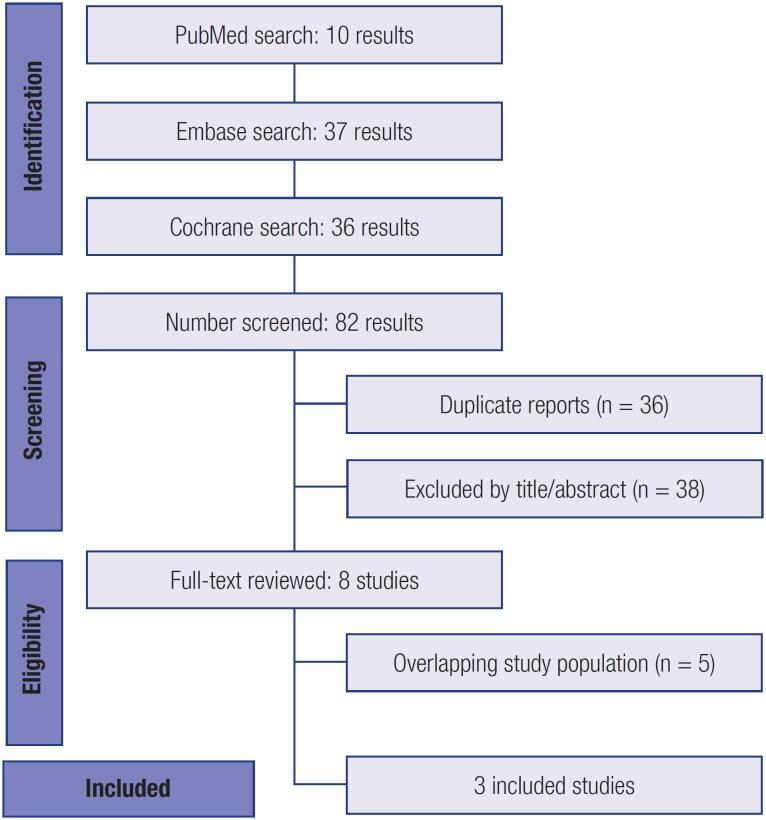
PRISMA flow diagram of study screening and selection.

As detailed in Figure 1, the initial search yielded 82 results. After removal of duplicate records and studies with and exclusion criterion based on title/abstract review, 8 studies remained and were fully reviewed for the inclusion and exclusion criteria. Ultimately, a total of 453 patients from 3 Phase 2 Randomized Controlled Trials were included in this systematic review and meta-analysis (4-6), with exclusion of five studies due to overlapping population. Insulin Icodec Once-Weekly was prescribed to 230 (50,77%) patients and Insulin Glargine U100 Once-Daily to 223 (49,22%) patients. Studies characteristics are described in Table 1. Mean follow-up ranged from 16 to 26 weeks.

**Table 1:** Baseline Characteristics of Included Studies in the Meta-analysis*

		ROSENSTOCK (4)	BAJAJ (6)	LINGVAY (5)
Population		TDM2 using oral hypoglycemic drugs	TDM2 using oral hypoglycemic drugs	TDM2 using oral hypoglycemic drugs
Intervention		Once weekly Icodec	Once weekly Icodec with Loading Dose	Once weekly Icodec Titration B
Control		Once daily Glargine U100	Once daily Glargine U100	Once daily Glargine U100
Study Design		RCT Phase 2 Double-blind	RCT Phase 2 Open-label	RCT Phase 2 Open-label
Follow-up		26 weeks (+5)	16 weeks (+5)	16 weeks (+5)
HbA1C for Inclusion		7%-9,5%	7%-10%	7%-10%
Target Fasting Glucose		70-108 mg/Dl	70-130 mg/dL	80-130 mg/dL
Previous Basal Insulin Therapy		No	Yes	No
Number of Patients				
Intervention		n = 125	n = 54	n = 51
	Control	n = 122	n = 50	n = 51
	Total	N= 247	N = 154	N = 205
Male Sex				
	Intervention	70 (56,0)	39 (72,2)	53,8
	Control	69 (56,6)	33 (66,0)	52,9
	Total	139 (56,3)	111 (72,1)	53,7
Age				
	Intervention	59,7 (8,2)	62,4 (7,2)	61,4 (8,0)
	Control	59,4 (9,5)	60,5 (7,9)	60,2 (8,1)
	Total	59,6 (8,9)	61,7 (7,8)	60,7 (8,3)
Diabetes Duration (years)				
	Intervention	10,5 (8,4)	13,8 (7,7)	9,2 (4,4)
	Control	8,8 (6,1)	14,8 (8,1)	11,8 (6,8)
	Total	9,7 (7,4)	15,1 (8,1)	10,1 (6,0)
Body Mass Index				
	Intervention	31,1 (4,9)	30,2 (4,3)	30,8 (3,8)
	Control	31,4 (4,4)	30,3 (5,0)	30,6 (4,7)
	Total	41,3 (4,6)	29,8 (4,5)	31,3 (4,5)
HbA1C (%)				
	Intervention	8,09 (0,7)	7,8 (0,7)	8,2 (0,9)
	Control	7,96 (0,6)	7,9 (0,7)	8,2 (0,8)
	Total	8,02 (0,6)	7,9 (0,7)	8,1 (0,8)
Fasting Plasma Glucose (mg/dL)				
	Intervention	182 (42)	142 (34)	177 (41)
	Control	180 (42)	148 (36)	168 (42)
	Total	181 (42)	144 (37)	175 (39)
Diabetes Complication				
	Intervention	27 (21,6)	NA	NA
	Control	22 (18,0)	NA	NA
	Total	49 (19,8)	NA	NA
Atherosclerotic Cardiovascular Disease				
	Intervention	8 (6,4)	NA	NA
	Control	5 (4,1)	NA	NA
	Total	13 (5,3)	NA	NA

* Absolute Number (Percentage) and Mean (Standard Deviation).

HBA1C: glycated hemoglobin; NA: not available; RCT: randomized controlled trial; TDM2: diabetes mellitus type 2.

**Figure 2:**
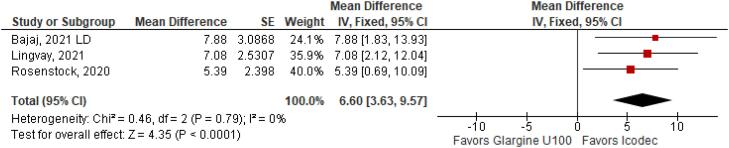
Mean Difference of Time with Glucose in Range (%) for Once-Weekly Icodec *vs.* Once-Daily Insulin Glargine U100.

**Figure 3:**
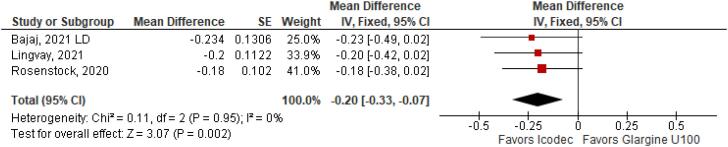
Mean Difference of HBa1C (%) Change from Baseline for Once-Weekly Icodec *vs.* Once-Daily Insulin Glargine U100.

With regards to the glycemic control, Time with Glucose in Range (MD 6.60% CI 3.63 to 9.57%; P < 0.0001; I^2^ = 0%; Figure 2) was higher in the Icodec group, as well as greater reduction in Glycated Hemoglobin (MD -0.20% CI -0.33 to -0.07%; P = 0.002; I^2^ = 0%; Figure 3) between patients who received Icodec and Glargine U100. There was no significant difference in Fasting Plasma Glucose (MD -2.62 mg/dL CI -7.80 to 2.57 mg/dL; P = 0.32; I^2^ = 0%; Figure 6) and body weight (MD 0.3 2kg CI -0.25 to 0.88 kg; P = 0.27; I^2^ = 0%; Figure 7) change from baseline. However, the insulin dose difference (MD 0,97 UI CI 0.76 to 1.18 UI; P = 0.02; I^2^ = 74%; Figure 8) was higher in the Icodec group.

**Figure 4:**
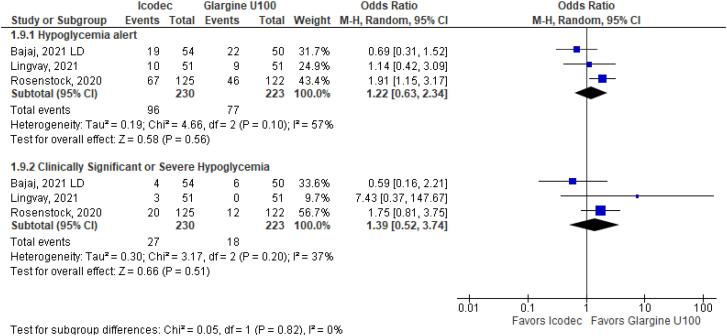
Odds Ratio of Hypoglycemia for Once-Weekly Icodec *vs.* Once-Daily Insulin Glargine U100.

**Figure 5:**
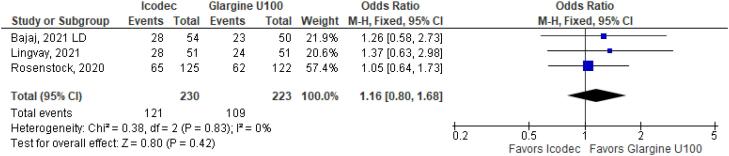
Odds Ratio of Any Adverse Outcome for Once-Weekly Icodec *vs.* Once-Daily Insulin Glargine U100.

**Figure 6:**
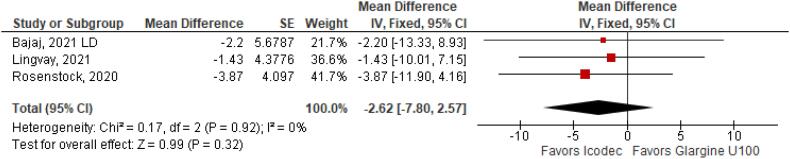
Mean Difference of Fasting Plasma Glucose (mg/dL) for Once-Weekly Icodec *vs.* Once-Daily Insulin Glargine U100.

**Figure 7:**
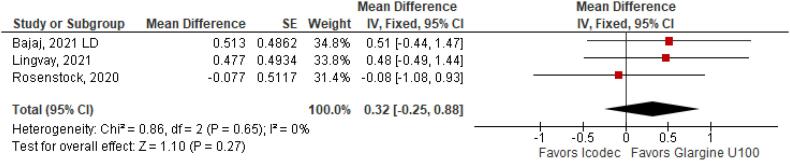
Mean Difference of Body Weight (kg) Change from Baseline for Once-Weekly Icodec *vs.* Once-Daily Insulin Glargine U100.

**Figure 8:**
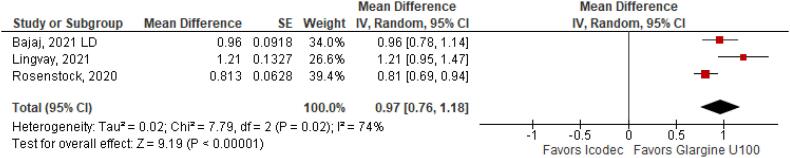
Mean Difference of Insulin Dose Difference (UI) for Once-Weekly Icodec *vs.* Once-Daily Insulin Glargine U100.

As for the safety endpoints, hypoglycemic alert (OR 1.22 CI 0.63 to 2.34; P = 0.56; I^2^ = 57%; Figure 4) and clinically significant or severe hypoglycemia (OR 1.39 CI 0.52 to 3.74; P = 0.51; I^2^ = 37%; Figure 4) had no significant difference in the once-weekly insulin icodec group, compared with once-daily insulin Glargine U100.

**Figure 9:**
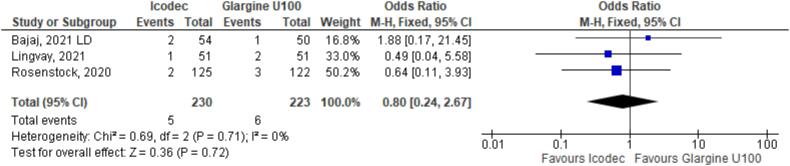
Forrest Plot of Once-Weekly Icodec *vs.* Once-Daily Insulin Glargine U100 for Serious Adverse Event.

**Figure 10:**
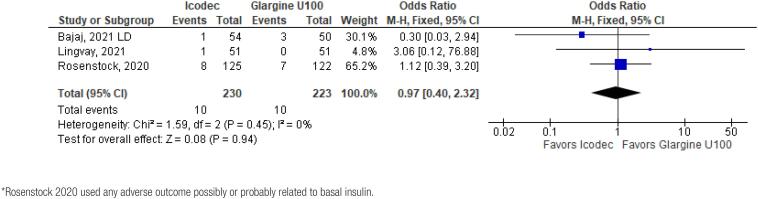
Forrest Plot of Once-Weekly Icodec *vs.* Once-Daily Insulin Glargine U100 for Any Adverse Event Probably related to Basal Insulin*.

**Figure 11:**
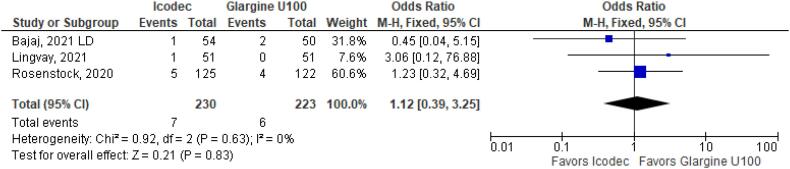
Forrest Plot of Once-Weekly Icodec *vs.* Once-Daily Insulin Glargine U100 for Injection Site Reaction.

**Figure 12:**
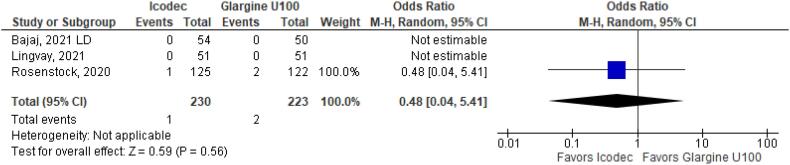
Forrest Plot of Once-Weekly Icodec *vs.* Once-Daily Insulin Glargine U100 for Hypersensitivity Event.

In addition, any adverse event (OR 1.16 CI 0.80 to 1.68; P = 0.42; I^2^ = 0%; Figure 5), serious adverse event (OR 0.80 CI 0.24 to 2.67; P = 0.72; I^2^ = 0%; Figure 9) and adverse event probably related to basal insulin (OR 0.97 CI 0.40 to 2.32; P = 0.94; I^2^ = 0%; Figure 10) showed no statistically significant difference. Injection site reaction (OR 1.12 CI 0.39 to 3.25; P = 0.83; I^2^ = 0%; Figure 11) and hypersensitity reaction (OR 0.48 CI 0.04 to 5.41; P = 0.56; Figure 12) also were not significant.

Table 2 outlines individual appraisal of each RCT included in the meta-analysis. Overall, all studies were deemed at low risk of bias. There was also no evidence of small-study effect by funnel plots. There was a symmetrical distribution of studies with similar weights around the meta-analysis point estimate (Figure 13–24).

**Table 2:** Risk of bias assessment of studies included in the meta-analysis

Bias Domain	ROSENSTOCK (4)	BAJAJ (6)	LINGVAY (5)
Randomization Process	Low	Low	Low
Deviations from Intended Interventions	Low	Low	Low
Missing Outcome Data	Low	Low	Low
Measurement of the Outcomes	Low	Low	Low
Selection of the Reported Result	Low	Low	Low
Overall Risk of Bias	Low	Low	Low

**Figure 13:**
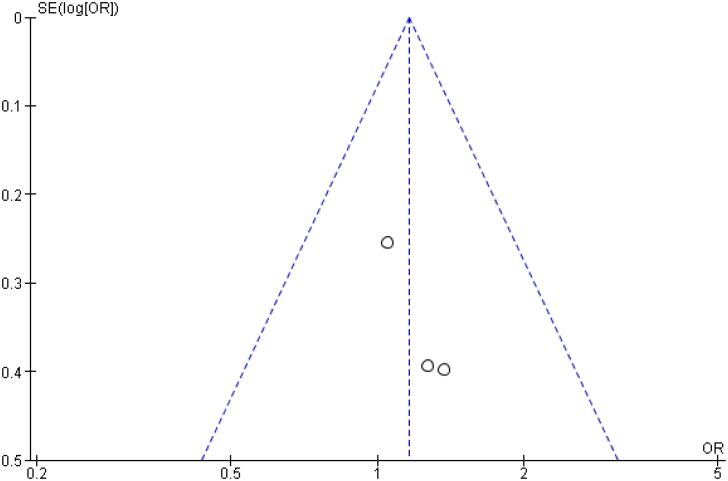
Funnel Plot of Once-Weekly Icodec *vs.* Once-Daily Insulin Glargine U100 for Any Adverse Outcome.

**Figure 14:**
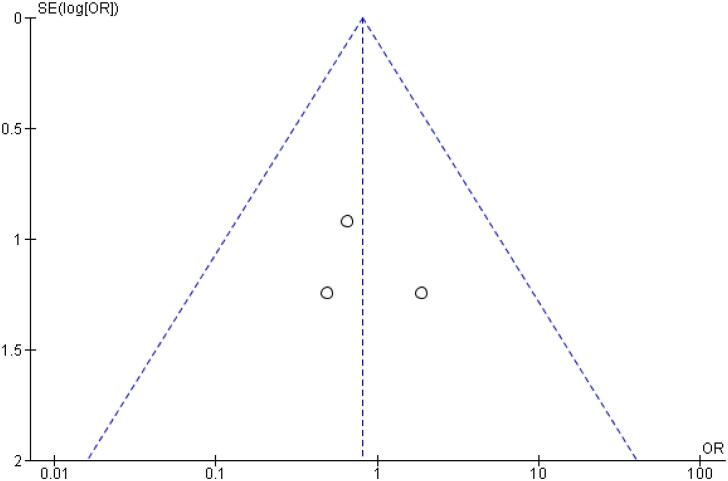
Funnel Plot of Once-Weekly Icodec *vs.* Once-Daily Insulin Glargine U100 for Serious Adverse Event.

**Figure 15:**
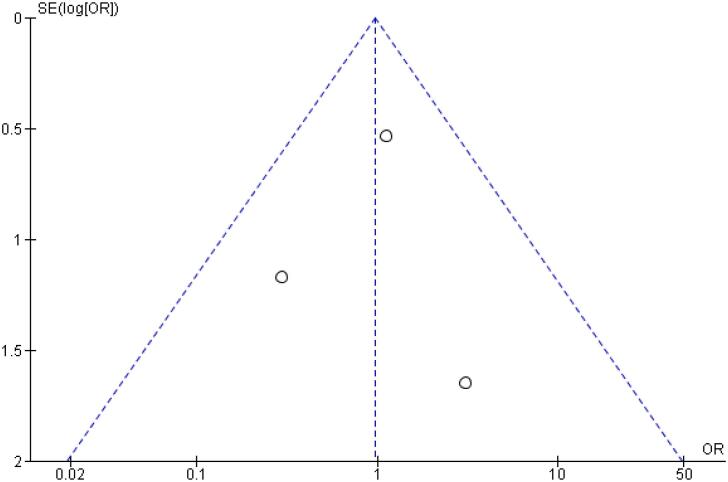
Funnel Plot of Once-Weekly Icodec *vs.* Once-Daily Insulin Glargine U100 for Any Adverse Event Probably related to Basal Insulin.

**Figure 16:**
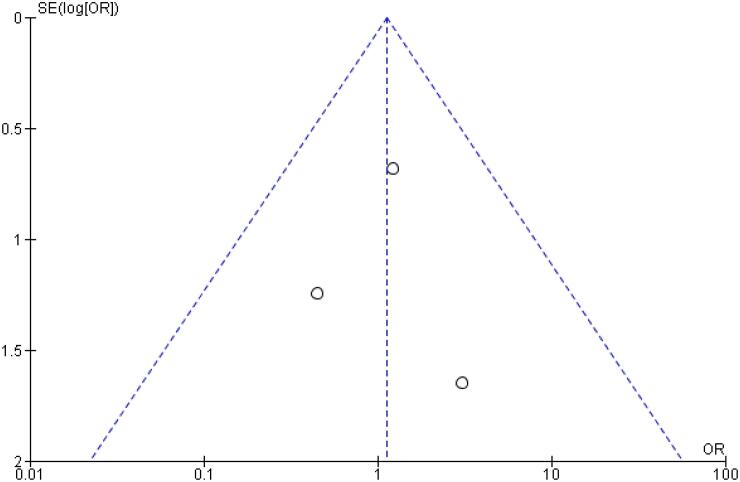
Funnel Plot of Once-Weekly Icodec *vs.* Once-Daily Insulin Glargine U100 for Injection Site Reaction.

**Figure 17:**
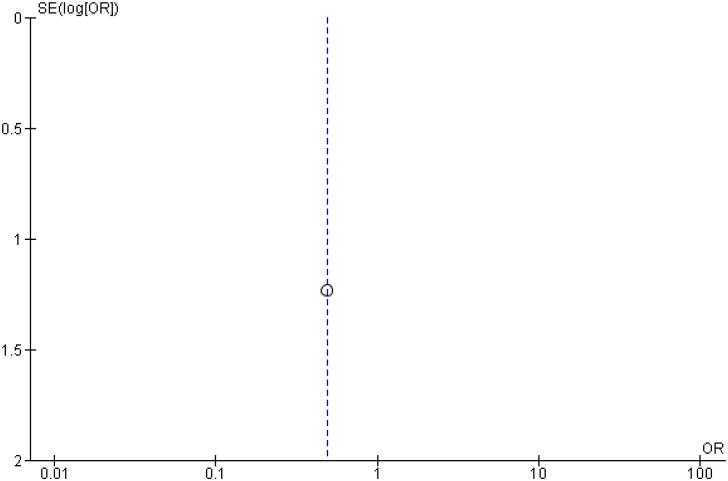
Funnel Plot of Once-Weekly Icodec vs. Once-Daily Insulin Glargine U100 for Hypersensitivity Reaction.

**Figure 18:**
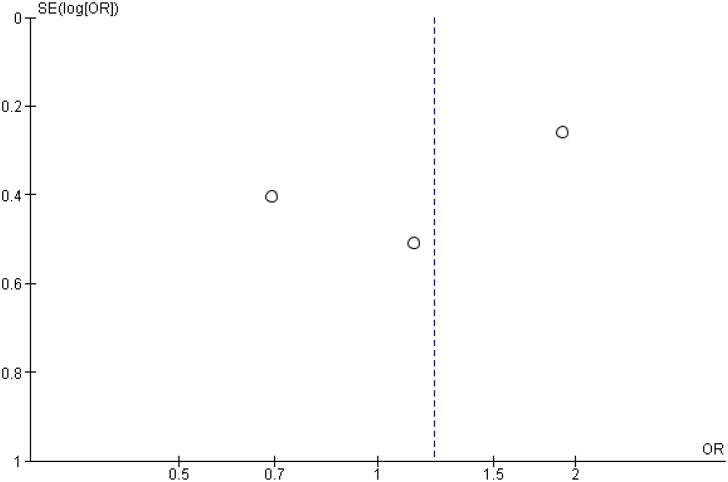
Funnel Plot of Once-Weekly Icodec *vs.* Once-Daily Insulin Glargine U100 for Hypoglycemia Alert.

**Figure 19:**
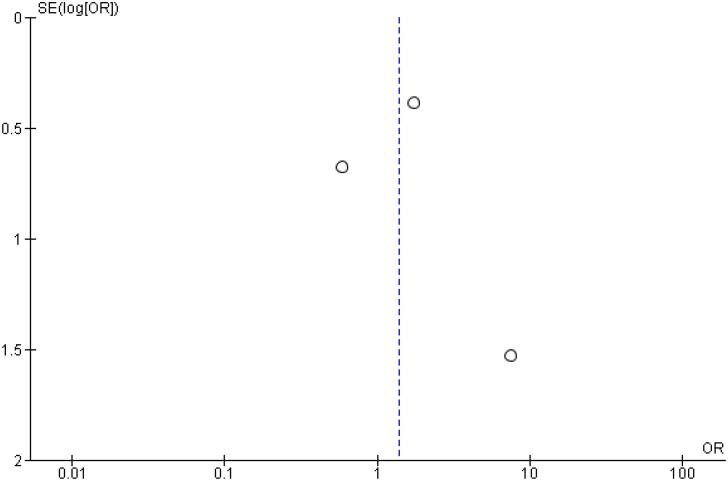
Funnel Plot of Once-Weekly Icodec *vs.* Once-Daily Insulin Glargine U100 for Clinically Significant or Severe Hypoglycemia.

**Figure 20:**
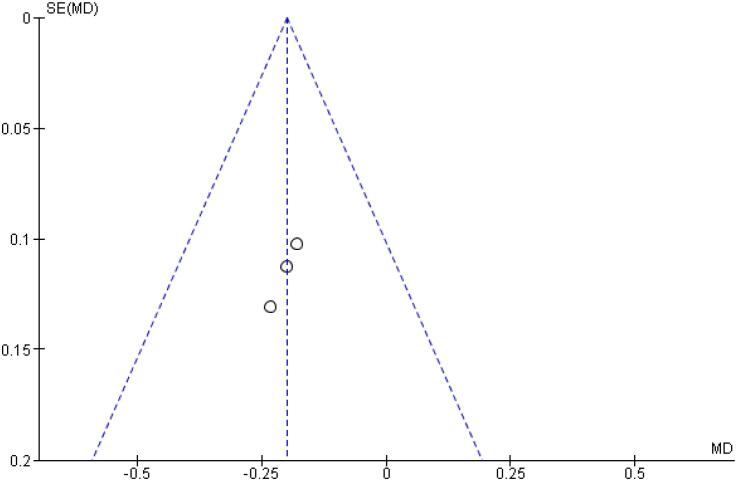
Funnel Plot of Once-Weekly Icodec *vs.* Once-Daily Insulin Glargine U100 for HBa1C (%) Change from Baseline.

**Figure 21:**
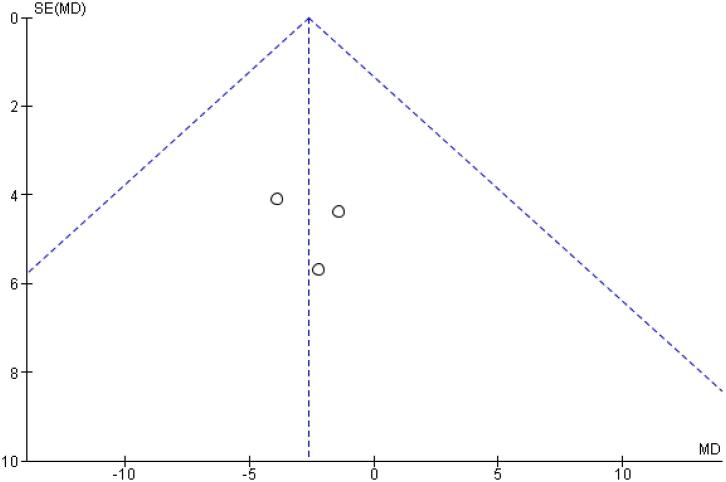
Funnel Plot of Once-Weekly Icodec *vs.* Once-Daily Insulin Glargine U100 for Fasting Plasma Glucose (mg/dL) Change from Baseline.

**Figure 22:**
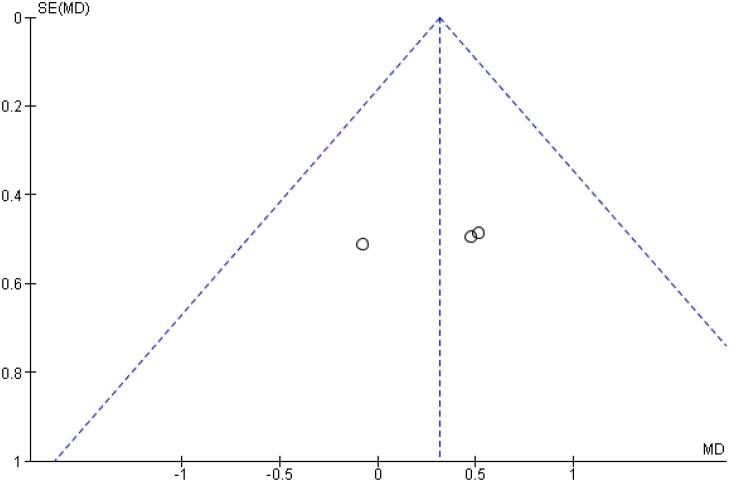
Funnel Plot of Once-Weekly Icodec *vs.* Once-Daily Insulin Glargine U100 for Body Weight (kg) Change from Baseline.

**Figure 23:**
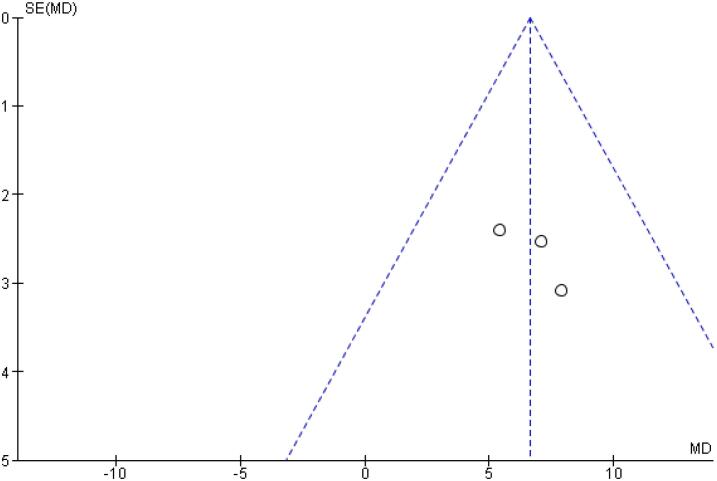
Funnel Plot of Once-Weekly Icodec *vs.* Once-Daily Insulin Glargine U100 for Time with Glucose in Range (%).

**Figure 24:**
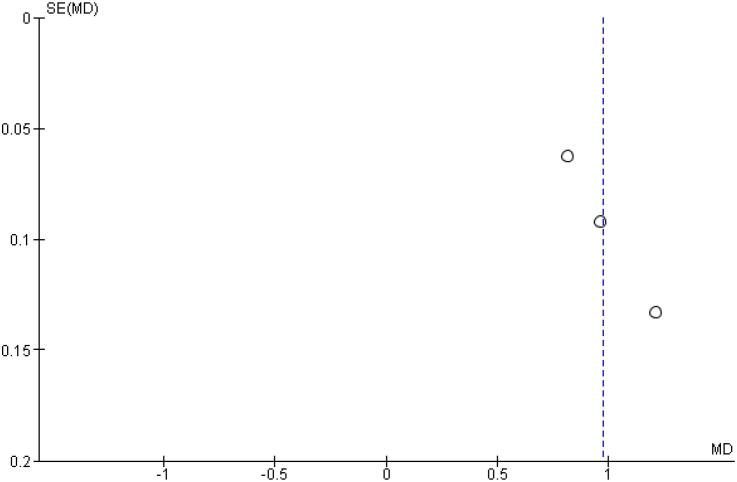
Funnel Plot of Once-Weekly Icodec *vs.* Once-Daily Insulin Glargine U100 for Insulin Dose Difference (UI).

**Figure 25:**
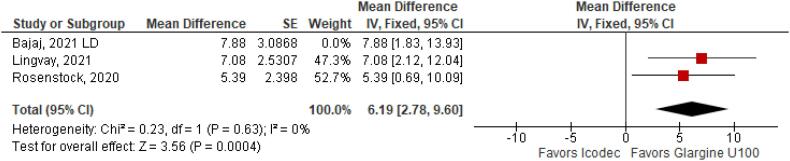
Mean Difference of Time with Glucose in Range (%) for Once-Weekly Icodec *vs.* Once-Daily Insulin Glargine U100 without Bajaj study (6).

**Figure 26:**
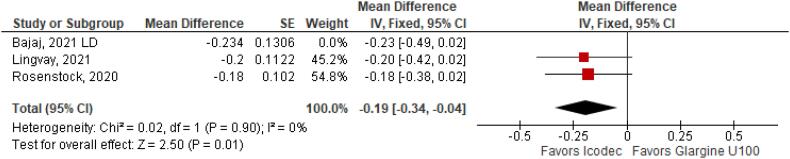
Mean Difference of HBa1C (%) Change from Baseline for Once-Weekly Icodec *vs.* Once-Daily Insulin Glargine U100 without Bajaj study (6).

**Figure 27:**
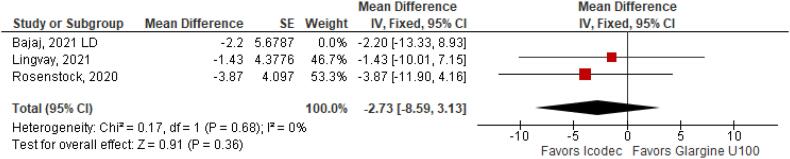
Mean Difference of Fasting Plasma Glucose (mg/dL) Change from Baseline for Once-Weekly Icodec *vs.* Once-Daily Insulin Glargine U100 without Bajaj study (6).

**Figure 28:**
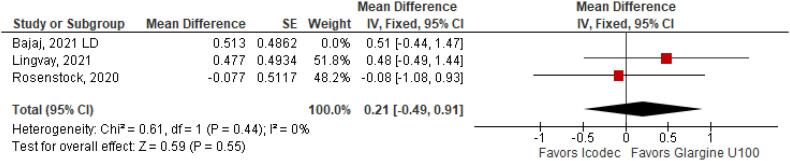
Mean Difference of Body Weight (kg) Change from Baseline for Once-Weekly Icodec *vs.* Once-Daily Insulin Glargine U100 without Bajaj study (6).

**Figure 29:**
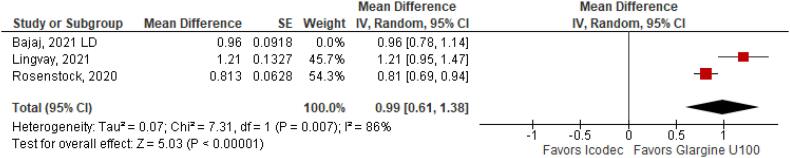
Mean Difference of Insulin Dose Difference (UI) for Once-Weekly Icodec *vs.* Once-Daily Insulin Glargine U100 without Bajaj study (6).

**Figure 30:**
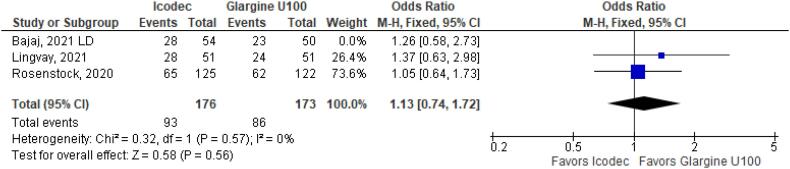
Odds Ratio of Any Adverse Outcome for Once-Weekly Icodec *vs.* Once-Daily Insulin Glargine U100 without Bajaj study (6).

**Figure 31:**
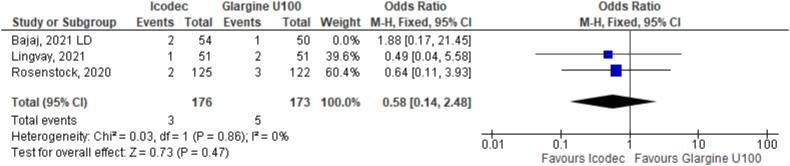
Odds Ratio of Serious Adverse Event for Forrest Plot of Once-Weekly Icodec *vs.* Once-Daily Insulin Glargine U100 without Bajaj study (6).

**Figure 32:**
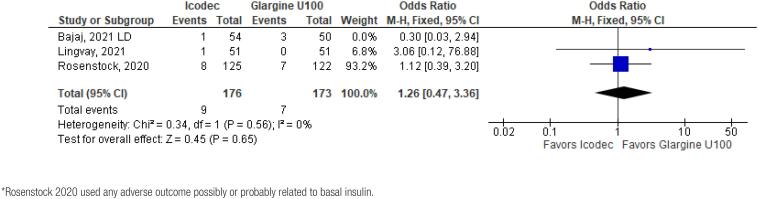
Odds Ratio of Any Adverse Event Probably related to Basal Insulin for Once-Weekly Icodec *vs.* Once-Daily Insulin Glargine U100 without Bajaj study (6)*.

**Figure 33:**
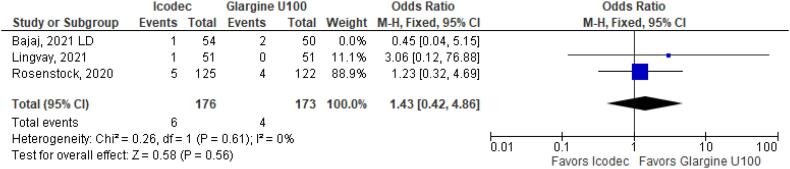
Odds Ratio of Injection Site Reaction for Once-Weekly Icodec *vs.* Once-Daily Insulin Glargine U100 without Bajaj study (6).

**Figure 34:**
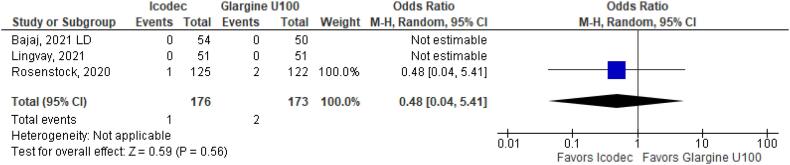
Odds Ratio of Hypersensitivity Reaction for Once-Weekly Icodec *vs.* Once-Daily Insulin Glargine U100 without Bajaj study (6).

**Figure 35:**
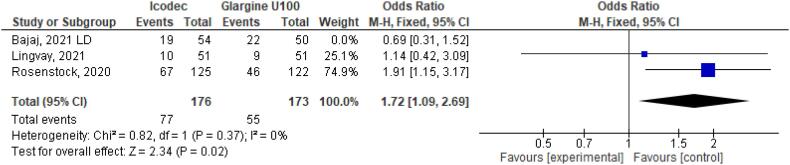
Odds Ratio of Hypoglycemia Alert for Once-Weekly Icodec *vs.* Once-Daily Insulin Glargine U100 without Bajaj study (6).

**Figure 36:**
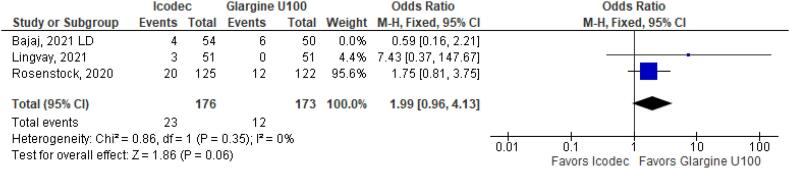
Odds Ratio of Clinically Significant or Severe Hypoglycemia for Once-Weekly Icodec *vs.* Once-Daily Insulin Glargine U100 without Bajaj study (6).

We conducted a sensitivity analysis, to assess the impact of individual studies in our results. When Bajaj study (6) was excluded from the analysis, Once-Weekly Insulin Icodec presented with higher odds ratio for Hypoglycemia Alert (OR 1.72 CI 1.09 to 2.69; P = 0.02; I^2^ = 0%; Figure 35). Nevertheless, Clinically Significant or Severe Hypoglycemia (OR 1.99 CI 0.96 to 4.13; P = 0.06; I^2^ = 0%; Figure 36) was still not significant after excluding Bajaj study (6). In addition, only Rosenstock study (4) contributed to hypersensitivity reaction outcome, since there were no events registered in Bajaj (6) or Lingvay (5) studies No more outcomes were altered by individual studies. Sensitivity analysis excluding Bajaj study is contained in Figures 25–36.

## DISCUSSION

In this systematic review and meta-analysis of 3 Phase 2 RCTs and 454 patients, we compared Once-Weekly Insulin Icodec with Once-Daily Insulin Glargine U100 in patients with Diabetes Mellitus Type 2. The main findings were as follows: (1) There was a 0.20% grater reduction of Glycated Hemoglobin from baseline in the Icodec group. (2) Insulin Icodec increased Time with Glucose in Range with a mean difference of 6.60%. (3) Patients in the Icodec group needed a higher weekly dose when compared with the Glargine group, with a mean difference of 0.97 UI. (4) There was no difference in regards to safety endpoints.

Insulin Icodec has stable pharmacokinetic and pharmacodynamic profiles supporting once-weekly administration. This can be attributed to a strong reversible albumin binding, slow receptor-mediated clearance and reduced enzymatic degradation (3,10). After subcutaneous injection, Icodec is absorbed and into the circulation and binds to albumin to form an essentially inactive depot. Thus, Icodec molecules slowly reaches insulin receptors to stimulate glucose lowering at target tissues. After each weekly injection, the pool of albumin-bound Icodec gradually increases, until steady state is reached after approximately 1 month when the full glucose lowering effect is achieved and insulin clearance matches administered insulin dose (3,10).

Concerning the frequency of injections, the reduction to weekly insulin could proportionate the same results observed with the advent of injectable once-weekly glucagon-like peptide 1 (GLP-1) (11). Studies comparing once-weekly and once-daily injectable GLP-1 showed increase in treatment adherence and patient satisfaction (11,12). The same benefits were seen in patients who never had injectable treatment and the ones previously treated who switched from daily to weekly injections (12). Although patient satisfaction is an important outcome for this new treatment, it was not evaluated in the included studies, but similar benefits can emerge from it. Furthermore, fewer injections might facilitate treatment initiation in patients with type 2 diabetes who have not previously taken insulin, by reducing clinical inertia and promoting better acceptance of insulin therapy (4).

The recommended mean TIR by the International Consensus is > 70% (13). In addition, it is known that each 5% incremental in TIR is associated with clinically significant benefits (13-16). After evaluating 1.440 participants, Beck and cols. (14) found that a reduction of 10% in TIR increased the hazard ratio of retinopathy in 64% and microalbuminuria in 40% (P < 0.001). Therefore, Once-Weekly Insulin Icodec presented with a notable 6.60% increase in TIR compared with Once-Daily Insulin Glargine U100, result that could indicate a potential reduction in diabetes complications.

The included studies were only Phase 2 RCTs, not powered to detect significant differences between treatments for any endpoint. This limited our data to a restrict sample of patients and excluding several subgroup of interest, such as patients with chronic kidney disease and patients using sulfonylureas. In this context, there is a need for larger Phase 3 RCTs to demonstrate the effect and safety profile of Insulin Icodec with greater quality of evidence. To this date, there are 22 RCTs registered on NIH clinical trials registry, 3 of them being the subject of study of the meta-analysis and 8 trials are already completed and awaiting the results (CT.gov).

Lingvay study (5) evaluated three different Icodec titration algorithms, divided by prebreakfast self-measured glucose target and weekly insulin dose adjustment. We included Icodec titration B in our analysis, since this group used a 28 unit/week adjustment and a prebreakfast target of 80-130 mg/dL which aligns with ADA recommendations (17). Moreover, titration C adopted a different target than Glargine U100 group, thus could add bias to the analysis, and titration A used a 21 unit/week adjustment, which differed from Rosenstock and Bajaj studies. In this context, a similar target of 70-130 mg/dL was used by Bajaj study (6), while Rosenstock study (4) adopted a tighter target, ranging from 70-108 mg/dL. Regarding insulin dose adjustment, all three studies applied a 28 unit/week adjustment.

Furthermore, Bajaj study (6) included patients with TDM2 already using basal insulin therapy, in contrast to Rovenstock (4) and Lingvay (5). As a result, there could be changes in the response to insulin therapy if a patient has been previously treated, thus could have influenced our results. In the Escalada study (18), insulin naive patients who initiated treatment with Glargine U100 had a slight increase in hypoglycemia, while insulin experienced patients who switched from any basal insulin to Glargine U100 showed less episodes of the outcome. Therefore, patients initiating treatment could be more susceptible to hypoglycemic episodes, due to an initial adapting period and the lack of knowledge in hypoglycemia prevention. Moreover, once weekly titration might difficult insulin dose adjustment, especially for naive patients, predisposing them to hypoglycemic episodes in the beginning of the treatment.

In addition, Bajaj study (6) compared two separate Icodec treatment regiments, with and without an initial loading dose. Since Insulin Icodec has a half-life of 1 week and reaches its steady state around 3-4 weeks, the cessation of a previous basal insulin could decompensate glycemic control (5,6). Therefore, an initial loading dose could minimize the impact of this transition. In this sense, we chose to include the loading dose group in our meta-analysis (6).

Early glucose control has been associated with a reduction of microvascular complications and MACE due to glycemic legacy (metabolic memory) (19). A 10-year post-intervention follow-up of the UKPDS showed cardiovascular benefit among patients with newly diagnosed T2DM who had been assigned to intensive glycaemic control (20). Knowing the highly prevalent clinical inertia for insulin initiation, with a delay of up to 4.9 years, a potential reduction in this time might lead to an improvement in these outcome (5). Furthermore, tighter goals might be sought in selected patients, such as short diabetes duration, long life expectancy and without evidence of cardiovascular disease, despite HbA1c recommended target being ≤ 7%. Therefore, the possibility of decreasing clinical inertia, associated with a greater reduction in HbA1c without an increase in hypoglycemia, could show great promise in TDM2 management (19).

Considering the reduction of only 0.2% in glycated hemoglobin and that the costs entailed to Once-Weekly Icodec were not disclosed, a cost benefit analysis should be conducted in a public health context (19). In this regard, Perez-Nieves and cols. evaluated the impact of basal insulin in patient adherence and heath costs. A reduction in total costs was observed with basal insulin due to higher adherence besides an initial large cost by reducing complications and hospitalizations over a period of 3 years (21).

Although methodological rigor was followed throughout the conduction if the study, the present review may have other limitations. Our research strategy included only studies in English and did not cover small data bases for the presence of RCT comparing insulin Icodec and Glargine U100 in patients with TDM2. Also, the authors of the original studies were not contacted for additional data. Nevertheless, we believe that none of this limitations would change the conclusions of this review.

In conclusion, Once-Weekly Insulin Icodec was associated with a small reduction in Glycated Hemoglobin, as well as higher TIR, with similar hypoglycemic events, in comparison to Glargine U100. The results from Phase 3 RCTs, as well as individual studies for patients with previous basal insulin therapy, could help measure the efficacy and safety of Insulin Icodec henceforth.
